# Effect of Deposit Scale on Mechanical Properties of In-Situ Alloyed CrCoNi Medium Entropy Alloys Formed by Directed Energy Deposition

**DOI:** 10.3390/ma17194795

**Published:** 2024-09-29

**Authors:** Pengsheng Xue, Dengke Liu, Zhongtang Gao, Guodong Wen, Yuan Ren, Xiangang Cao

**Affiliations:** 1 School of Mechanical Engineering, Xi’an University of Science and Technology, Xi’an 710054, China; dengkeliu1996@163.com (D.L.); zhongtanggao@xust.edu.cn (Z.G.); gdwen@xust.edu.cn (G.W.); 2 School of Mechanical Engineering and Automation, Northeastern University, Shenyang 110819, China; 3 State Key Laboratory for Mechanical Behavior of Materials, School of Materials Science and Engineering, Xi’an Jiaotong University, Xi’an 710049, China; ry715822@stu.xjtu.edu.cn

**Keywords:** medium entropy alloy, additive manufacturing, in-situ alloying, deposit scale, mechanical properties

## Abstract

Directed energy deposition (DED), as an additive manufacturing technology, has shown unique advantages in multi-material additive manufacturing and remanufacturing. In this study, two types in-situ alloyed CrCoNi medium entropy alloys that have thin-walled structures with different thicknesses (T1 and T2) were manufactured by the DED process, and the mechanisms of differences in relative density, microstructure, and mechanical properties at different heights were systematically analyzed. In terms of microstructure, the T1 and T2 samples along the building direction exhibit significant differences in crystallographic orientation, grain size, and dislocation density, which are related to the local temperature gradient differences caused by the scanning path and heat accumulation. In terms of mechanical properties at different heights of the two types of thin-walled structures, the yield strength is higher but the elongation is lower at the bottom position of sample, while the yield strength is lower but the elongation is higher at the middle and top positions. The differences of mechanical properties at different heights of the T1 and T2 samples are related to the microstructure and relative density. This finding provides new insights for the design and performance analysis of complex thin-walled structures formed by additive manufacturing.

## 1. Introduction

Additive manufacturing (AM), also known as 3D printing technology, has brought revolutionary changes to the manufacturing industry due to its near net forming advantages [1]. As a major laser metal AM method, directed energy deposition (DED) has shown unique advantages in part-repair and multi material part-forming [2]. At present, materials such as stainless steel [3], nickel-based high-temperature alloys [4,5], and titanium alloys [6] have been widely used in AM. Medium/high entropy alloys, as an emerging material with excellent properties in recent years, have gradually been used in AM processes [7]. As a subset of CoCrFeNiMn HEA, the strength and toughness of CrCoNi medium-entropy alloy (MEA) exceed most HEAs and multi-phase alloys at present, and it has broad application prospects in cryogenic fields such as deep space exploration and liquid hydrogen [7].

At present, research on AM of face centered cubic (FCC) phase medium/high entropy alloys mainly focuses on analyzing the effects of process parameters on microstructure and mechanical properties, and on improving their mechanical properties by changing material composition [8,9]. Li et al. [10] obtained selective laser melted (SLM) CoCrNi samples at different volumetric energy densities by changing laser power and scanning speed. It is found that the differences in mechanical properties of the samples are related to the changes in melt pool boundaries and crystallographic orientation caused by different process parameters. Ge et al. [11] also found that there is a credible parameter window for SLM CrCoNi samples. Yi et al. [12] also conducted a tensile properties analysis on SLM CrCoNi samples after optimizing process parameters, elucidating the activation and interaction mechanisms of dislocations, stacking faults, twinning-induced plasticity, and transformation-induced plasticity. At the same time, it is found that there are sequential twinning and phase transformation pathways in the printed CoCrNi sample. In addition, Pan et al. [13] found that hot cracks are eliminated, and grains are refined during the SLM CoCrNi process by adding appropriate TiC. Meanwhile, the segregation of C leads to the formation of Cr_23_C_6_ and core-shell structure (TiO_2_/TiC) nanoprecipitates along the grain boundaries, which helps to increase the mechanical properties. Bi et al. [14] also studied the mechanical anisotropy of DED CoCrNi samples through experiments and molecular dynamics and found that the formation of strong crystallographic textures is the reason for the mechanical anisotropy.

As mentioned above, although there have been many studies on AM CoCrNi (as shown in Table 1), there are still some issues that need to be resolved. On the one hand, current research mainly focuses on the use of pre-alloyed powders, while fabricate parts using elemental powder mixtures by AM process can achieve high-throughput design of materials and reduce manufacturing costs; but research is insufficient. On the other hand, current research focuses on the effects of process parameters on the microstructure and mechanical properties of formed samples, while there is still a lack of study on the microstructure and mechanical properties at different forming geometric dimensions. Therefore, two main issues are studied in this work. The first is to analyze the relative density and microstructure of in-situ alloyed CoCrNi samples by DED process at different thicknesses and building heights. The second is to elucidate the mechanism of mechanical property differences induced by these factors.

## 2. Materials and Experimental Procedure

### 2.1. Materials and Experimental Details

**Figure 1:**
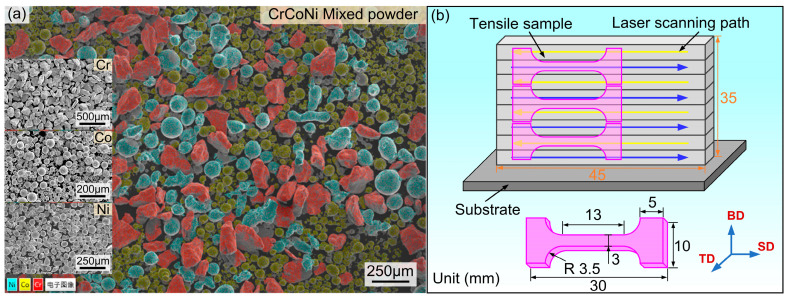
(**a**) The SEM image and EDS map of mixed powder; (**b**) sample size and tensile direction.

The raw materials used in the experiment are Cr, Co, Ni powders, and their average sizes are 200 μm, 75 μm, 80 μm, respectively (The supplier is Beijing Yanbang New Material Technology Co., Ltd., Beijing, China). The mixed powder as depicted in Figure 1a was obtained by ball milling for 30 min at speed 200 rpm, and the ball to powders ratio is 5:1. It can be seen that the various powders are uniformly distributed by an energy dispersive spectrometer (EDS) analysis. The mixed-powder was dried for 2 h at 393 K in order to remove moisture. The manufacturing process of the sample was carried out on a five-axis additive and subtractive hybrid CNC machining center (SVW80C-3D) equipped with a 2000 W fiber laser; and the process parameters used are as follows: laser power P = 900 W, scanning speed Vs = 6.0 mm/s, Z-axis lift ΔZ = 0.4 mm, laser spot diameter 3 mm. Two types thin-walled structures with different thicknesses (T1 = ~2.3 mm, T2 = ~4.7 mm) were formed through single-track and multi-track accumulation, respectively. The dimensions of the formed part and tensile sample, as well as the sampling positions of the tensile sample are shown in Figure 1b.

### 2.2. Microstructural Characterization

The sample was first ground with 240–2000 mesh sandpaper, and then polished with 2.5 μm diamond suspension and 0.12 μm SiO_2_ colloidal oxide polishing suspension. The relative density analysis of the sample was obtained by analyzing the binarized optical microscopy images of pores through Image Pro Plus software. The X-ray diffraction (XRD) analysis was performed on X-ray diffractometer (SmartLab 9 KW, Rigaku, Woodlands, TX, USA) with Cu Kα radiation and scanning within 2θ = 20°–110°. The EDS of the sample was observed through field emission scanning electron microscopy (FE-SEM, Zeiss Ultra Plus, ZEISS, Oberkochen, Germany). The electron backscattered diffraction (EBSD) measurement of the sample was performed on focused ion beam scanning electron microscope (FIB-SEM, Crossbeam 550, ZEISS, Oberkochen, Germany).

### 2.3. Analysis of Mechanical Properties

The tensile properties of the samples were carried out at a displacement rate of 0.3 mm/min in a universal testing machine (DNS-10) at room temperature, and each group of samples was repeated at least three times. The fracture morphology of the tensile sample was observed by field emission scanning electron microscopy to analyze the failure mechanism.

## 3. Results and Discussions

### 3.1. Porosity

**Figure 2:**
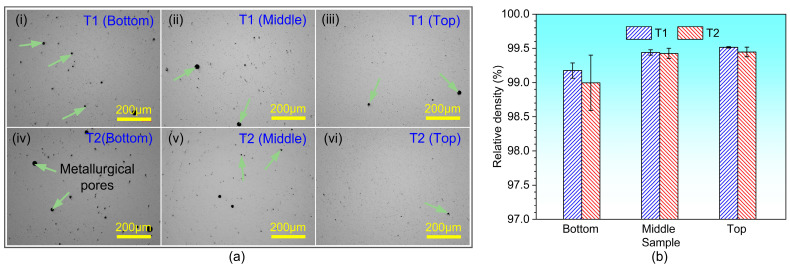
(**a**) Optical microscopic images of pores, (**b**) Relative density.

Figure 2a,b shows the optical microscopy images of pores and relative density at different height positions (Top: 25 mm, Middle: 15 mm, Bottom: 5 mm) of thin-walled samples with different thicknesses. From Figure 2a, it can be seen that spherical metallurgical pores appeared in two types of thin-walled samples. At the same time, it is found that compared to the metallurgical pores in the middle and top positions, the size of that in bottom position is larger. In addition, as shown in Figure 2b, the relative density of the bottom position of two types thin-walled samples is lower, while the relative density of the middle and top positions is higher and there is no significant difference. The larger metallurgical pores in the bottom position of two types of thin-walled samples are mainly related to its faster cooling rate. In the DED process, the cladding layer near the substrate has a faster cooling rate at the beginning of printing due to the small amount accumulation of heat [15]. Therefore, during the forming process, the gas carried by the powder itself or protective gas is trapped in the molten pool and appeared in the solidified melt. There are fewer pores in the middle and top positions of the sample mainly because the cooling rate is reduced due to the accumulation of heat in the bottom cladding layer.

### 3.2. XRD Analysis

**Figure 3:**
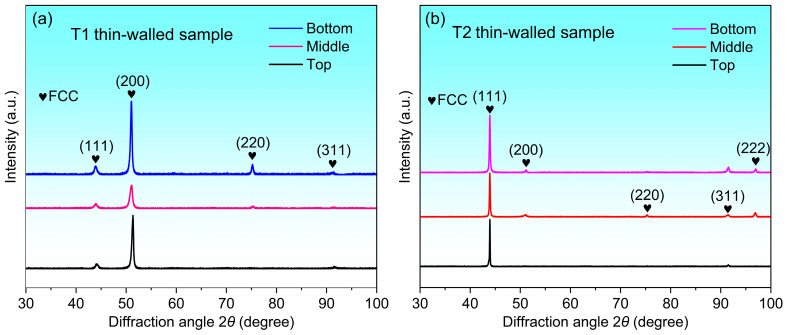
XRD analysis of thin-walled structures with different thicknesses.

The XRD analysis of the thin-walled samples with different thicknesses is shown in Figure 3. It can be seen that the XRD patterns of all samples show a single FCC phase structure, which indicates that the DED process maintains the original crystal structure of the powder. In the T1 thin-walled sample, the diffraction peak intensity of (200) is obviously higher than that of other diffraction peaks, which indicates that there is an obvious crystallographic orientation. There is also a significant (111) diffraction peak intensity in T2 sample, but its preferred orientation is significantly different from that of the T1 sample. The difference between (200) and (111) preferred orientation is mainly related to the change of the local temperature field of the melt pool caused by the laser scanning strategy [16,17], which will be discussed in detail in the EBSD analysis.

### 3.3. Microstructures

**Figure 4:**
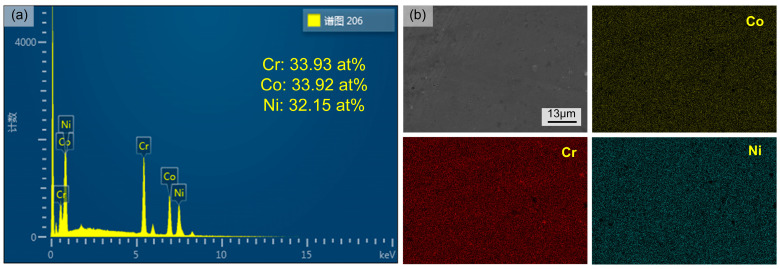
(**a**) EDS analysis of in-situ alloyed CrCoNi thin-walled structures; (**b**) SEM image of in-situ alloyed CrCoNi thin-walled structures.

As shown in Figure 4, the EDS analysis showed that the proportion of each element basically met the design requirements, and obvious macro-segregation of elements is not apparent, indicating that the in-situ alloying of CrCoNi was achieved by the DED process using the element powder mixtures.

**Figure 5:**
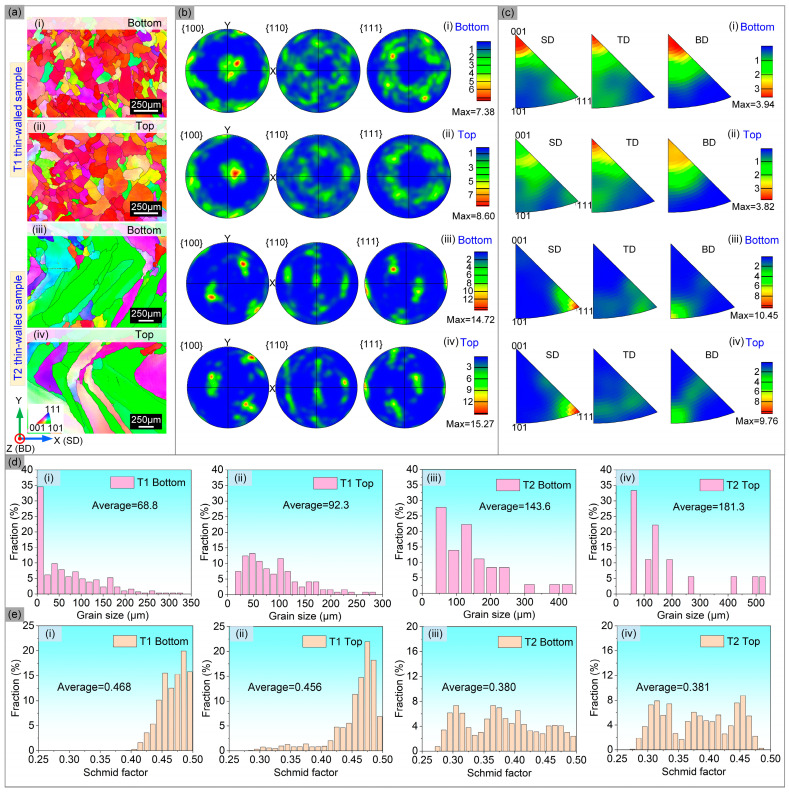
EBSD analysis of thin-walled structures with different thicknesses. (**a**–**c**) Inverse pole figure (IPF) and pole figures; (**d**) grain size distribution; (**e**) Schmid factor distribution.

**Figure 6:**
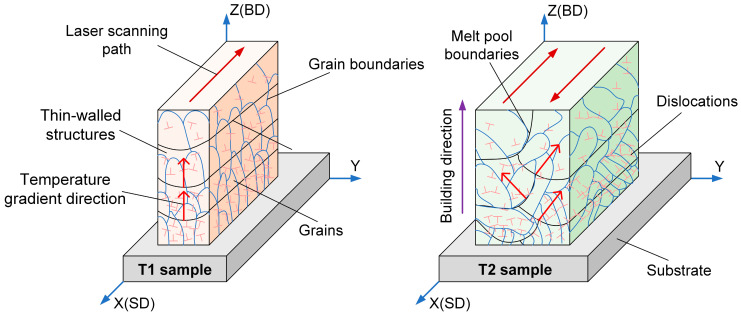
Schematic diagram of temperature gradient, microstructure, and dislocation density.

Figure 5 shows the EBSD analysis of the thin-walled samples with different thicknesses. It can be seen that <001> crystallographic orientation along the building direction appeared in the T1 sample and an obvious peak intensity appeared on the {100} pole figure, indicating that a cubic texture similar to a single crystal appeared in the T1 sample [18]. At the same time, similar equiaxed grains in the X–Y plane also confirm the directional growth of grains along the building direction [19]. However, in the X–Y plane of the T2 sample, the long columnar grains of about 45° to the coordinate axis appeared, and it also showed a strong <111>//SD crystallographic orientation. In short, crystallographic orientation did not change significantly at different building heights, while crystallographic orientation changed significantly at different thicknesses. This is closely related to the changes in temperature field caused by the laser scanning path [20], and the formation mechanism, which is shown in Figure 6. In the forming process of the T1 sample, the cladding layers only accumulated along the building direction and did not show lateral migration, so the local temperature gradient is mainly along the building direction. The next cladding layer would be completely parallel and covered on the bottom layer, which would result in the epitaxial growth of cylindrical grains along the building direction and through multiple cladding layers [21,22]. In the forming process of the T2 sample, the cladding layer also appeared side-lap, so that the local temperature gradient produced a component perpendicular to the building direction, and the grains are grown in the direction of about 45° from the building direction.

The analysis of grain size and the Schmid factor at the top and bottom positions of the thin-walled samples with different thicknesses are shows in Figure 5d,e, and it can be seen that the grain size at the bottom position is smaller than that at the top position. There is no significant difference in the Schmid factor along the building direction, and the Schmid factor along the scanning direction showed a smaller value in the T2 samples. The increase of grain size along the building direction is mainly related to the cooling rate, and the cooling rate is larger due to a small amount of heat accumulation at the beginning of printing. The heat accumulation in the middle and top positions of sample tends to be stable, which form a stable and small cooling rate. The Schmid factor difference is mainly related to the preferred crystallographic orientation. Compared with the <001> preferred orientation in the scanning direction (SD) of the T1 sample, the <111> preferred orientation in the SD direction of the T2 sample is not conducive to the movement of the dislocation slip, so it shows a hard orientation with the smaller Schmid factor.

### 3.4. Mechanical Properties

#### 3.4.1. Tensile Properties

**Figure 7:**
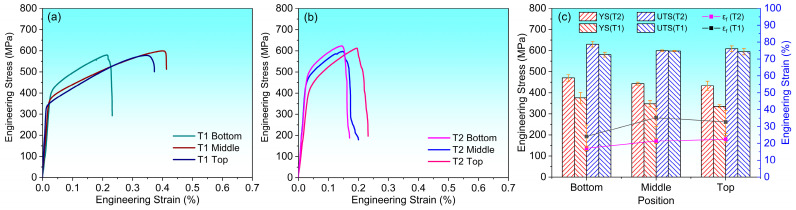
Mechanical properties of the thin-walled structures with different thicknesses. (**a**,**b**) Engineering stress-strain curves; (**c**) Histogram of microhardness, yield strength (YS), ultimate tensile strength (UTS) and elongation (εf).

The representative stress-strain curves of different heights in the thin-walled samples with different thicknesses are shown in Figure 7a,b, and the yield strength (YS), ultimate tensile strength (UTS), and elongation (*ε_f_*) values are shown in Figure 7c and Table 2. It can be seen that in the thin-walled samples with two types thicknesses, the bottom position of the sample shows a higher YS but a lower elongation, while the mechanical properties of the middle and top positions are the opposite to those of the bottom position and have no significant differences. At the same heights, the T2 sample has a higher YS but a lower elongation than the T1 sample, and the bottom position of the T2 sample shows the highest YS and the lowest elongation.

The differences in YS ($\sigma_{y}$) of samples with different heights and thicknesses are mainly related to grain size, dislocation density and crystallographic orientation, which can be expressed by Equation (1) [23]:

(1) $$\sigma_{y}=\sigma_{0}+\sigma_{g}+\sigma_{d}$$

where $\sigma_{0}$ is the intrinsic strength (or lattice friction stress) [24], $\sigma_{g}$ is the contribution of grain size to YS [25], and $\sigma_{d}$ is the contribution of dislocation density to YS [26].

The contribution of grain size to YS can be expressed by the Hall–Petch relationship [27]:

(2) $$\sigma_{g}=kd^{-1/2}$$

where k is the Hall–Petch slope of the material, and d is the mean grain size. In this study, we use the value of k form CrCoNi, which is $265\;MPa\;\mu m^{1/2}$ according to Ref. [28].

The effect of initial dislocation density and crystallographic orientation on YS can be measured by Equation (3) [26]:

(3) $$\sigma_{d}=M\alpha Gb\rho^{1/2}$$

where M is the Taylor factor [29], G is the shear modulus of CrCoNi (G=87 GPa), α is the empirical constant (α=0.2 for FCC alloy [30]), b is the Burgers vector (b=0.253 nm [31]) and ρ is total dislocation density [32].

The total dislocation density ρ can be obtained by the WH method shown in Equations (4) and (5) [33].

(4) $$\beta \cos\theta =\frac{K\lambda }{d}+(4\sin\theta )\cdot \varepsilon$$

(5) $$\rho =2\sqrt{3}\cdot \varepsilon /(db)$$

where β is true XRD peak broadening, θ is Bragg angle, constant K=0.9 [34], and the wavelength of Cu Kα radiation λ=0.15405 nm.

The main factors affecting YS at different heights for the thin-walled samples with different thicknesses are grain size and dislocation density, because there is no obvious difference in the preferred orientation along the building direction. As can be seen from Table 3, the grain sizes of the T1 and T2 samples along the building direction increase from 68.8 μm and 143.6 μm to 92.3 μm and 181.3 μm, respectively. The dislocation density is decreased from $4.642*10^{13}\;m^{-2}$ and $1.914*10^{14}\;m^{-2}$ to $3.239*10^{13}\;m^{-2}$ and $1.068*10^{14}\;m^{-2}$, respectively. The difference of dislocation density along the building direction is mainly related to the cooling rate. The research shows that the faster cooling rate produces large thermal residual stress, which increases the dislocation density [35]. Therefore, under the combined effect of grain size and dislocation density, the YS of the sample shows a decreasing trend along the building direction.

There are obvious differences in grain size, dislocation density and preferred orientation for the thin-walled samples with different thickness at the same building height (as shown in Figure 6). Compared with the T1 sample, T2 has a larger grain size but a larger YS, which can be explained by dislocation density and preferred orientation. The Schmid factor value is closely related to crystallographic orientation. As can be seen from Figure 5e, the Schmid factor value of the T1 and T2 samples at the same building height are 0.468/0.456 and 0.380/0.381, respectively. Compared with the T1 sample, T2 has larger dislocation density and preferred orientation with smaller Schmid factors. The large initial dislocation density acts as a barrier, which increases the resistance of dislocation slip during deformation [36]. However, the smaller Schmid factor value along the tensile direction have a hard orientation with a larger Taylor factor, so the dislocation slip is not easy to carried out when loading along the <111> crystallographic plane. Thus, the larger YS of the T2 sample compared to the T1 sample at the same building height is due to the larger initial dislocation density and the <111> preferred orientation along the SD direction, while the grain size has less effects.

The variation of the elongation at different heights is mainly related to the relative density, while the difference of the elongation at different thicknesses is mainly related to the crystallographic orientation. At the same thickness, there is no obvious difference in grain morphology and crystallographic orientation at different heights, but the relative density has certain difference. In the process of tensile deformation, the larger pores distributed in the bottom position are easy to become the center of micropores [7], resulting in easier crack propagation and poor plasticity. At the same building height, the crystallographic orientation of samples with different thicknesses is significantly different. Strong <001>//SD preferred orientation existed in T1 sample, while strong<111>//SD orientation appeared in T2 sample. When loading along the SD direction, <111> orientation is not conducive to dislocation slip, which increases YS, but decreases plasticity.

#### 3.4.2. Fracture Analysis

**Figure 8:**
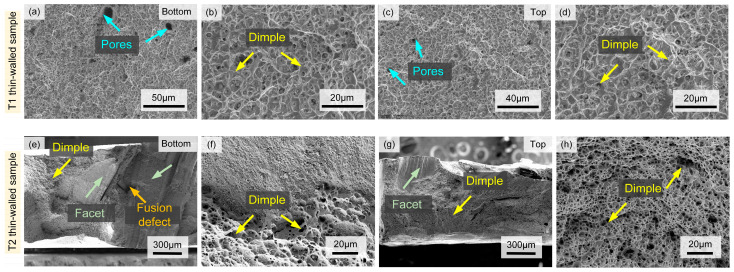
(**a**,**c**,**e**,**g**) SEM image of fracture; (**b**,**d**,**f**,**h**) high magnification SEM image of fracture.

The representative fracture morphologies of the top and bottom positions of thin-walled samples with different thicknesses are shown in Figure 8. It can be seen that fracture is relatively flat and shows a large number of dimples in the T1 sample, which indicates that the fracture mode is a ductile fracture [37]. At the same time, the fracture shows fewer pores and a larger dimple in the top position than those in the bottom position, which is consistent with its good plasticity. However, the fracture of the T2 sample is uneven and shows a mixture of dimple and facet, which indicates that the fracture mode is mainly ductile fracture. Compared with the fracture at the bottom position of the sample, the fracture at the top position has fewer facet and pores due to better plasticity.

## 4. Conclusions

In this study, an in-situ alloyed CrCoNi medium-entropy alloys that have thin-walled samples with two different thicknesses were fabricated by directed energy deposition. The relative density, microstructure, and tensile properties of the samples at different heights were systematically analyzed. The main conclusions are as follows:

(1) Spherical metallurgical pores appeared in thin-walled samples with two different thicknesses. The relative density of the bottom position of the sample is the lowest, while the relative density of the middle and top positions is higher and there is no significant difference.

(2) The <001> preferred orientation along the building direction and similar equiaxed grain morphology in the X-Y plane appeared in the T1 sample, while the strong <111>//SD preferred orientation and long columnar grains of about 45° to the coordinate axis in the X–Y plane existed in the T2 sample. The increase in grain size and decrease in dislocation density along the building direction were observed in the samples with two different thicknesses, while there are no significant differences in the Schmid factor related to crystallographic orientation.

(3) The bottom position of the T1 and T2 samples showed higher yield strength but lower elongation, while the middle and top positions had smaller yield strength with no significant differences and higher elongation. At the same height, compared to the T1 sample, the T2 sample had higher yield strength but lower elongation. The bottom position of the T2 sample showed the highest yield strength and the lowest elongation. These differences in mechanical properties are related to grain size, crystallographic orientation, and dislocation density.

## Figures and Tables

**Table 1:** Summary of research on additive manufacturing of CrCoNi alloy.

Materials	Technology	Feature	References
CoCrNi	SLM	Differences in mechanical properties under different volumetric energy densities.	[10]
CoCrNi	SLM	Elucidated the interaction mechanisms of dislocations, twinning induced plasticity, and phase transition induced plasticity.	[12]
CoCrNi	SLM	Analyzed the effects of TiC addition on hot cracking, grain size, and mechanical properties.	[13]
CoCrNi	DED	The mechanical anisotropy of the sample was studied through experiments and molecular dynamics simulations.	[14]

**Table 2:** The mechanical properties of thin-walled structures with different thicknesses at different heights.

Samples	YS (MPa)	UTS (MPa)	εf (%)
T1 Top	335.93	594.68	32.63
T1 Middle	347.63	597.93	35.21
T1 Bottom	375.58	580.55	24.13
T2 Top	433.27	608.75	22.46
T2 Middle	443.29	599.55	21.35
T2 Bottom	470.42	629.83	17.12

**Table 3:** Relative density and microstructure analysis of T1 and T2 samples at different heights.

Samples	Relative Density	Grain Size	Dislocation Density	Schmid Factor	Preferred Orientation
T1 Top	99.175%	92.3 μm	$3.239*10^{13}\;m^{-2}$	0.456	<001>
T1 Bottom	98.997%	68.8 μm	$4.642*10^{13}\;m^{-2}$	0.468	<001>
T2 Top	99.514%	181.3 μm	$1.068*10^{14}\;m^{-2}$	0.381	<101>
T2 Bottom	99.446%	143.6 μm	$1.914*10^{14}\;m^{-2}$	0.380	<101>

## Data Availability

The original contributions presented in the study are included in the article, further inquiries can be directed to the corresponding authors.
